# Vulnerability of individual fish to capture by trawling is influenced by capacity for anaerobic metabolism

**DOI:** 10.1098/rspb.2015.0603

**Published:** 2015-08-22

**Authors:** Shaun S. Killen, Julie J. H. Nati, Cory D. Suski

**Affiliations:** 1 Institute of Biodiversity, Animal Health and Comparative Medicine, College of Medical, Veterinary and Life Sciences, University of Glasgow, Graham Kerr Building, Glasgow G12 8QQ, UK; 2 Department of Natural Resources and Environmental Sciences, University of Illinois, 1102 S Goodwin Avenue, Urbana, IL 61801, USA

**Keywords:** fisheries-induced evolution, metabolic rate, aerobic scope, anthropogenic selection, conservation physiology

## Abstract

The harvest of animals by humans may constitute one of the strongest evolutionary forces affecting wild populations. Vulnerability to harvest varies among individuals within species according to behavioural phenotypes, but we lack fundamental information regarding the physiological mechanisms underlying harvest-induced selection. It is unknown, for example, what physiological traits make some individual fish more susceptible to capture by commercial fisheries. Active fishing methods such as trawling pursue fish during harvest attempts, causing fish to use both aerobic steady-state swimming and anaerobic burst-type swimming to evade capture. Using simulated trawling procedures with schools of wild minnows *Phoxinus phoxinus*, we investigate two key questions to the study of fisheries-induced evolution that have been impossible to address using large-scale trawls: (i) are some individuals within a fish shoal consistently more susceptible to capture by trawling than others?; and (ii) if so, is this related to individual differences in swimming performance and metabolism? Results provide the first evidence of repeatable variation in susceptibility to trawling that is strongly related to anaerobic capacity and swimming ability. Maximum aerobic swim speed was also negatively correlated with vulnerability to trawling. Standard metabolic rate was highest among fish that were least vulnerable to trawling, but this relationship probably arose through correlations with anaerobic capacity. These results indicate that vulnerability to trawling is linked to anaerobic swimming performance and metabolic demand, drawing parallels with factors influencing susceptibility to natural predators. Selection on these traits by fisheries could induce shifts in the fundamental physiological makeup and function of descendent populations.

## 1. Introduction

Humans are effective predators, and selective harvest of animals by humans probably represents one of the strongest drivers of evolutionary change for wild animal populations [1–3]. Hunting and fishing are selective processes which often remove individuals that, under normal circumstances, may have the highest reproductive potential [2,4,5]. Available evidence suggests that selection by harvest can lead to genetic change within wild populations for specific traits [1,5–7]. For example, the preferential harvest of large individuals from fish populations has caused evolutionary changes to key life-history traits, including growth trajectories, reproductive investment and size at maturity [8–11], a phenomenon termed ‘fisheries-induced evolution’ (FIE) [8,12–14]. In addition to varying in their *desirability* as a target for harvest, however, individual animals also differ in their *vulnerability* to harvest [13,15,16]. Interestingly, artificial selection on traits that affect harvest vulnerability can sometimes act in parallel with natural selection when selecting on specific traits. For example, several recent studies indicate that individual variation in behaviour can influence vulnerability to harvest. In some fish species, for example, bolder or more active individuals have a higher risk of predation and may also be more susceptible to capture by recreational and commercial fisheries ([17–19], but see [20]).

In contrast to the effects of behavioural traits on harvest vulnerability, there is almost nothing known regarding the role of individual physiology in determining which animals are most likely to be harvested [21]. Specifically, traits related to energy requirements (e.g. metabolic rate) and locomotory performance (e.g. aerobic scope (AS)) vary among individuals of the same species and are likely to influence vulnerability through a variety of mechanisms [22]. In commercial fishing operations, for example, fish often escape capture by employing behaviours linked to aerobic and anaerobic capacity [23,24]. During fishing by trawling, schools of fish are herded ahead of an oncoming net [25]. At the trawl mouth, fish attempt to maintain station relative to the moving net; those that fatigue or swim relatively slowly drop back into the net extension and finally into the codend, where they are retained [23,26]. Fish can escape by swimming faster than the moving net, or swimming above or below the outside of the trawl mouth, making it possible that better swimming fish may be more likely to escape capture. The speed of net tows is generally species-specific and selected to coincide with the upper limits of the sustained swimming ability of the targeted species [25]. Ultimately, the size of fish retained within a trawl is limited by mesh size and devices that can allow smaller fish to escape [22]. However, fish that have a higher swimming performance or anaerobic capacity may have a higher probability of avoiding net entry [24,25].

Importantly, many whole-animal physiological traits linked to locomotory ability, and potentially under selection by forms of harvest such as commercial trawling, are also related to biologically and ecologically important processes [21]. For example, due to associations between minimal metabolic rate (standard metabolic rate in ectotherms; SMR) and growth [19,20], direct or indirect selection by fisheries on metabolic rate could be linked to observed changes in growth rates and maturation size at the population level [21]. AS is the difference between an animal's minimal and maximal rate of aerobic metabolism, and represents the total capacity for simultaneous oxygen-consuming functions above maintenance, including growth and locomotion. In fishes, AS is correlated with swimming endurance, maximum sustainable speed and recovery rate after exhaustive exercise [22,23], all of which may be relevant to a fish's ability to evade capture by trawling. AS is also theorized to be associated with a number of ecological phenomena and the ability of ectotherms to cope with environmental stressors [24–26]. Similarly, rapid bursts of anaerobically powered movements are commonly used by animals to either escape predators or capture prey [10], and may also play a key role in escape from active harvest methods such as trawls. Increased anaerobic capacity could allow an individual to spend more time ahead of the trawl before fatigue, or allow an individual to quickly move towards the edge of the trawl while maintaining forward velocity.

The use of laboratory experiments with small-bodied schooling fish species has several advantages for the study of FIE relative to studying wild, commercial stocks. Such alternate species have been used to simulate size-selective harvest [27,28] and can also be easily subjected to scaled-down simulations of the same gears and practices used in commercial fisheries and used in experimental manipulations not possible with larger fish [18]. Although there are acknowledged limitations [28], the use of surrogate species, and particularly those that have not previously experienced selective pressure through harvest [18,28], is a valuable starting point for addressing concerns regarding fisheries practices that would be impossible with full-scale fisheries operations. For example, we still have no knowledge of whether the propensity to be captured by trawling is repeatable, which is a requirement for a trait to be a potential target for either natural or harvest-induced selection [29].

To date, no work has been performed to define whether physiological traits play a role in determining individual vulnerability to trawling. To examine this issue, we used forced swimming within a laboratory swim tunnel, outfitted with a simulated trawl net, to define the importance of a suite of measures of metabolism and swim performance in predicting vulnerability to trawl capture. We also assessed whether vulnerability to trawling is repeatable. We used wild-caught individuals of the common minnow, *Phoxinus phoxinus*, as this is a small, shoaling fish that lives in association with the substrate, making this species a suitable representation of commercially targeted benthopelagic species that are harvested while schooling (e.g. Atlantic cod *Gadus morhua* and haddock *Melanogrammus aeglefinus*). We hypothesized that repeatable variation in vulnerability would be related to metabolic and performance traits: the least catchable fish will be those with a relatively high AS, swimming performance and anaerobic capacity because these fish will be most able to avoid the net during forced swimming; fish that are most catchable were expected to show lower AS, swimming performance and anaerobic capacity.

## 2. Material and methods

### (a) Animals

Approximately 600 common minnows *P. phoxinus* were collected by large dip-nets from the River Kelvin near Glasgow, Scotland. They were first herded along shallow side-channels to improve collection efficiency and minimize the likelihood of any collection bias. Common minnows grow to a maximum size of 14 cm [30] and are not subjected to any commercial or recreational harvest. Collected minnows were held in the laboratory in two aerated stock tanks (100 × 40 × 30 cm) supplied with recirculating, UV-treated water at 14.5°C. All holding tanks, including those described for group holding during trawling simulations, received identical water, and all contained numerous plastic plants and pebble substrate. Minnows were fed daily ad libitum using a mixture of commercial feed (pellets and flakes), bloodworms and *Daphnia*, and kept on a 12 L : 12 D photoperiod. Fish were held in the laboratory under these conditions for eight months before experiments began and showed no visible signs of stress or disease at the time of testing. Of all fish in the stock tanks, 43 were used in the study (mean body mass = 1.03 ± 0.06 g; mean total length = 4.94 ± 0.09 cm).

### (b) Measurement of metabolic traits

Fish were removed from their stock tanks with dip-nets. To minimize the chance of selection bias due to ease of capture, a fish was first haphazardly targeted by eye, then pursued until retained within the dip-net. To reduce variation in body size among experimental fish, an effort was made to select individuals that were approximately 4–6 cm total length.

Maximum metabolic rate (MMR) was measured after exhaustive exercise by manually chasing individual fish in a circular tank (50 cm diameter) with a water depth of 10 cm [31]. This method assumes that maximal rates of oxygen uptake are achieved during the recovery from the bout of exhaustive anaerobic exercise [32,33]. After complete exhaustion, fish were transferred into individual cylindrical 75 ml glass respirometry chambers connected to an intermittent stopped-flow respirometry system [31,34]. Water oxygen content was quantified once every 2 s using a Firesting 4-channel oxygen meter and associated sensors (PyroScience GmbH, Aachen, Germany). Respirometers were placed within an aerated, rectangular, temperature-regulated water bath (14.5°C ± 0.1°C; 50 l), and were shielded from disturbance and direct lighting by an opaque plastic blind. Water mixing within each respirometer was achieved with a peristaltic pump that moved water through the chamber and around an external circuit of gas-impermeable tubing. Every 8 min, an automated flush pump would switch on for 2 min to flush chambers with fresh water, and, when switched off, sealed the respirometers to allow the decrease in oxygen content to be analysed to indicate the rate of oxygen uptake. To estimate MMR, we calculated rates of oxygen uptake for each 3 min time interval throughout the first 30 min of recovery immediately following the exhaustive exercise; MMR (mg O_2_ h^−1^) was taken as the highest rate of aerobic metabolism during this period.

After measurement of MMR, fish remained in the same respirometry chambers overnight to allow the measurement of routine metabolic rate (RMR) and SMR. Individuals were then removed from the respirometer at around 09.00 the following day, having remained in the respirometers for approximately 20 h in total. They were then lightly anaesthetized using benzocaine, given a unique combination of coloured VIE elastomer tags (Northwest Marine Technology Inc.) to allow individual identification when in shoals, measured for wet mass and total length, and then placed into common holding tank for 4 days before continuing with the remainder of the study. Whole-animal SMR (mg O_2_ h^−1^) was estimated as the lowest 10th percentile of measurements taken throughout the measurement period [35]; RMR (mg O_2_ h^−1^) was measured as the mean level of oxygen uptake. The first 5 h of confinement in the chambers was excluded from analyses of SMR and RMR because the oxygen consumption of fish was often elevated at this time. Excess post-exercise oxygen consumption (EPOC) for each individual was estimated by calculating the area under the exponential recovery function, above RMR, until the time at which fitted values were equal to individual RMR [36]. EPOC represents the increase in oxygen consumption above routine levels occurring during recovery from a bout of exhaustive anaerobic exercise and represents the anaerobic capacity of an animal [37]. AS was calculated as the difference between MMR and SMR.

### (c) Trawling simulations

Two days before beginning trawling simulations, the marked fish were taken from their common holding tank and randomly assigned to one of six groups (seven fish per group for 42 fish in total) that were held in separate holding tanks (38 × 38 × 21 cm).

Trawling simulations were performed in a 30-l Steffensen-type swim tunnel (Loligo systems, Tjele, Denmark) designed to exercise fish at controlled speeds in non-turbulent water with a uniform velocity profile, thermoregulated at 14.5° ± 0.5°C. The working section of the tunnel was 45 long × 14 wide × 14 high cm [35]. A false bottom was fabricated out of dense foam material (5 cm deep), and tightly fit into the floor of the working section of the tunnel. This was required so that a groove could be cut into the false floor to aide in securing the deployed net. Further, with the net frame below the false floor, the profile of the deployed net (i.e. the angle of the net relative to the ground) was more realistic relative to an actual demersal trawl. Finally, the false floor also prevented fish from bracing themselves against the net frame to avoid swimming. Prior to starting experiments, the swim tunnel was calibrated using a vane wheel flow meter (Flowtherm NT, Höntzsch, Waiblingen, Germany), with both the false bottom and the simulated trawl net in place (in the deployed and non-deployed positions; see below), to define water velocities.

The design of trawls can vary depending on several factors including the depth of deployment and the species targeted. In this study, we simulated processes occurring during the critical phase of capture common to virtually all trawl designs—the pursuit of fish as they attempt to hold station and resist entry at the trawl mouth (electronic supplementary material, video S1). For trawl simulation, groups of seven marked fish (previously assigned to stable groupings described above) were introduced into the swim tunnel and allowed 30 min to acclimate to a velocity that averaged 0.5 body lengths (BLs) per second (based on mean total length of the entire population of all minnows in the study). Following this acclimation period, water velocity was gradually increased to 38 cm s^−1^ (approximating an average of 8 BL s^−1^) over a period of approximately 30 s. Preliminary trials demonstrated that this velocity was the minimum that induced non-steady ‘burst-and-coast swimming’ for all individuals, a gait involving the recruitment of anaerobic fast-twitch glycolytic fibres [38,39]. Throughout this increase in speed, a simulated trawl net was moved forward from the rear of the swim tunnel, encouraging fish towards the anterior portion of the swim tunnel. The net was then secured behind the school of fish in a groove cut into the foam floor 14 cm from the front of the working section of the tunnel (i.e. the fish had a 14 cm space in which they could swim without entering the net). The mouth of the net consisted of a 3.5-mm steel wire frame (14 cm wide × 17 cm high), fitted with a custom-designed net made from stretched nylon (3 mm mesh). The net contained a cylindrical extension section, supported by a plastic ring insert (3.6 cm diameter) half way down its length. The net was approximately 28 cm long when fully deployed, including a bulged codend at its downstream end. This conical net produced a visual cue that induced an avoidance response by the fish, encouraging them to swim upstream of the deployed net (electronic supplementary material, video S1). We prevented fish from moving around the secured net by sizing the net to span the entire width of the swim tunnel and to extend slightly above the surface of the water. Fish were unable to swim around, under or above the net, and were forced to enter the net (and eventually codend) if they reduced or ceased swimming (electronic supplementary material, video S1). It was assumed that an increased propensity to spend time ahead of the net would be associated with an increased likelihood of moving away from or around the net (i.e. the fish would be less ‘catchable’), if this option were available to the fish (as occurs during full-scale trawls).

With the net deployed, fish were left to swim at 38 cm s^−1^ for a total of 10 min, during which time all swimming activity in front of and in the mouth of the net was recorded with a high-definition video camera (Sony Handycam, HDR XR260; electronic supplementary material, video S1). The duration of actual trawls is variable and can last in the range of 30–120 min [40]. However, whether or not fish enter the net or escape at the trawl mouth is generally decided within a few minutes of swimming ahead of the trawl [22,41]. At the end of the 10 min period, all fish were removed from the tunnel and returned to their holding tanks. Fish that fell back into the net during trials were undamaged, with the exception of one fish that showed minor fin abrasion and so was replaced within its group with another minnow for the second trial. Metabolic traits for this replacement fish (MMR, SMR, RMR, AS and EPOC) were measured following the trawling simulations. All fish quickly recovered after the trawling simulations and behaved normally within several minutes of returning to their holding tanks. Minnows were given a small meal and left overnight until an identical trawling simulation was repeated the next day, using identical groupings of fish.

Video recordings of trials were later analysed and the behaviour of each individual within each group was recorded using Solomon Coder (v. 14.05.18; Budapest, Hungary). For each fish, we quantified the total time spent in the net throughout the 600 s trial, defined as any time the snout of fish moved beyond the mouth of the net.

### (d) Measurement of swimming performance

Following the trawl simulations, all fish were allowed to recover in their group holding tanks for 3 days before individual quantification of aerobic and anaerobic swimming performance using a constant acceleration test [35,42]. Individual fish were placed in the swim tunnel (the false bottom was still present but the net was removed) and given 30 min to acclimate at a velocity of 5 cm s^−1^ (approx. 1 BL s^−1^, based on the mean total length of all fish in the study). Water velocity was then gradually increased at a rate of 2.5 cm s^−1^ min^−1^. A high-definition video camera (Sony Handycam, HDR XR260) recorded fish swimming patterns from above to identify the gait transition speed (*U*_gt_). Gait transition occurs when fish switch from steady aerobic swimming with slow-twitch oxidative muscle fibres, to non-steady, anaerobic burst swimming. A fish was deemed to have begun reliance on burst-and-coast swimming when it displayed four burst-and-coast events within a 1-min increment. The corresponding speed at the beginning of this increment was taken as *U*_gt_ and was considered the maximum aerobic swim speed of each fish [38]. Water current speed was increased past *U*_gt_ until the fish fell against the retaining grid at the posterior end of the tunnel and did not resume swimming for 5 s; the speed at which this occurred was taken as each individual's maximum swim speed (*U*_max_) and was used as an indicator of maximal anaerobic performance.

### (e) Statistical analyses

All analyses were performed with SPSS statistics v. 20.0 (SPSS Inc. and IBM, Chicago, IL, USA). The level of significance for all tests was *a* = 0.05. The repeatability of total time in net across trials was quantified as the intraclass correlation coefficient (ICC) using the methods of Lessells & Boag [43]. The influence of metabolic traits on vulnerability to capture by trawl was assessed using a linear mixed-effects (LME) model. The model was constructed using total time in the net as the response variable, and log SMR, log AS, log EPOC and log body mass as explanatory variables. The influence of swimming performance on capture by trawl was also assessed using an LME, but in this case, log *U*_max_, log *U*_gt_ and total length were used as explanatory variables. Fish ID and trawling test group were included as random effects on both the slope and intercept of both models. Separate LMEs were used for the effects of metabolic traits and performance indices because metabolic traits must be corrected for any effect of body mass on whole-animal metabolism (and so mass is included as a covariate), while absolute swimming performance can be affected by BL and so total length was included as a covariate. All main effects were kept in the models, even when non-significant. This was done to control for the effects of body size (mass or length, as appropriate) as well as to obtain relative parameter estimates of all explanatory variables traits of interest (i.e. metabolic traits and measures of swimming performance). The normality, linearity and homoscedasticity of residuals of the models were verified by inspection of residual-fit plots.

## 3. Results

### (a) Repeatability of vulnerability to trawling

**Figure 1.**
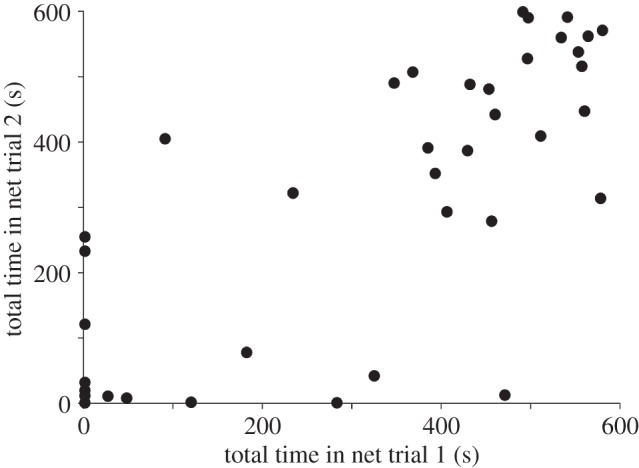
Total time spent in the net during two simulated trawling trials, each lasting 10 min (600 s) in duration. Repeatability = 0.77; Pearson correlation between trials: *r* = 0.76, *p* < 0.0001. Each data point represents one individual fish.

The total time fish spent within the net during the simulated trawl showed strong repeatability (figure 1), with 77% (ICC = 0.77) of the variance in the dataset being attributable to consistent differences among individuals between trials, as opposed to variation within individuals.

### (b) Factors affecting vulnerability to trawling

**Figure 2.**
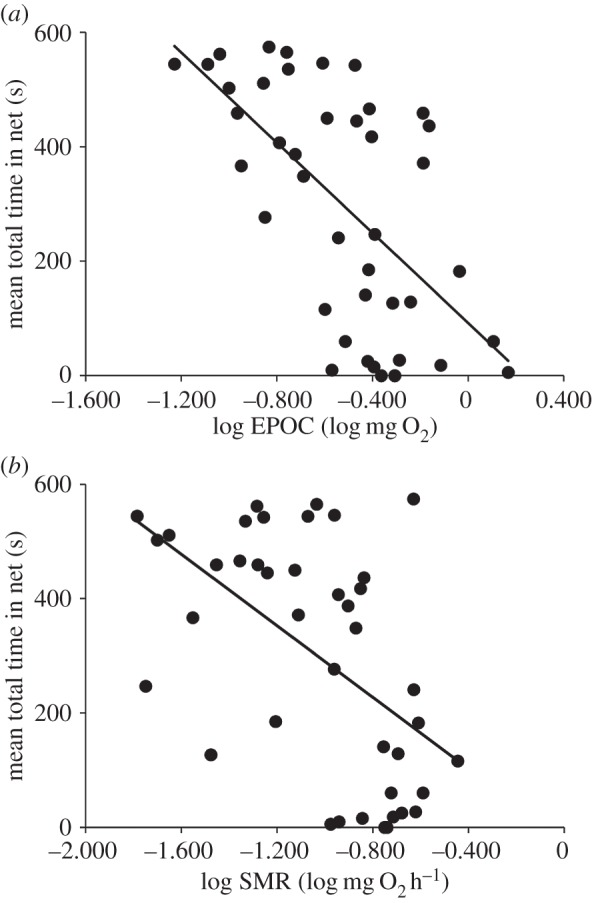
Relationships between time spent in the simulated trawl net and (*a*) anaerobic capacity, and (*b*) standard metabolic rate (SMR), for common minnows. Anaerobic capacity was measured using excess post-exercise oxygen consumption (EPOC). Regression lines are as follows: (*a*) *y* = 91.74 + (−393.97*x), *r*^2^ = 0.39, *p* < 0.0001; (*b*) *y* = −22.88 + (−312.62*x), *r*^2^ = 0.29, *p* = 0.0003. Each data point represents the mean net time for one individual fish taken over two trials (see Material and methods). However, linear mixed models included the data for each trial. Corrections for body size are included in the model presented in table 1.

**Figure 3.**
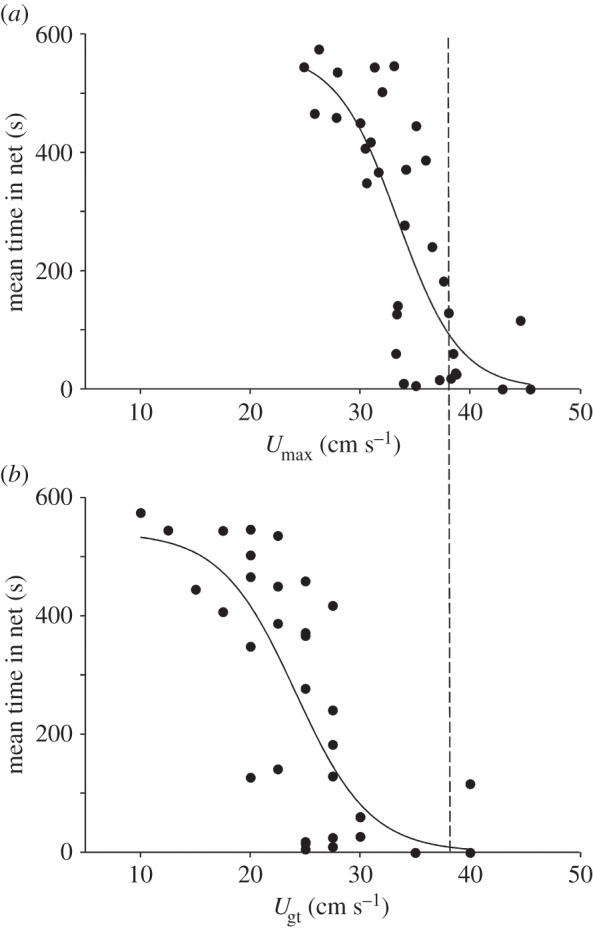
The relationships between (*a*) maximal anaerobic swimming speed (*U*_max_) and (*b*) maximum aerobic swim speed (*U*_gt_) and mean total time spent in the simulated trawl net by common minnows. The dotted horizontal line represents the water current speed in the trawling simulation. Relationships are as follows: (*a*) 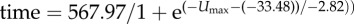, *r*^2^ = 0.63, *p* < 0.0001; (*b*) 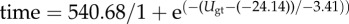, *r*^2^ = 0.57, *p* < 0.0001. Each data point represents the mean net time for one individual fish taken over two trials (see Material and methods). However, linear mixed models included the data for each trial. Corrections for body size are included in the model presented in table 1.

Fish that had a higher anaerobic capacity (as measured by EPOC) and higher SMR spent less time in the trawl net (figure 2 and table 1). Similarly, fish with a higher *U*_max_ and *U*_gt_ spent less time in the net (figure 3 and table 1). These effects persisted after accounting for the effects of body mass on metabolic traits, and the effects of total length on indices of swimming performance (table 1). AS did not affect time spent in the net. There were several correlations among metabolic traits and measures of swimming performance (electronic supplementary material, table S2).

**Table 1.** Linear mixed-effects models for the influence of metabolic traits and swimming performance on time spent in the net during repeated simulated trawls. SMR, standard metabolic rate (mg O_2_ h^−1^); AS, aerobic scope (mg O_2_ h^−1^); EPOC, excess post-exercise oxygen consumption (anaerobic capacity; mg O_2_); *U*_gt_, gait transition speed (cm s^−1^); *U*_max_, maximum speed (cm s^−1^).

model	term	numerator d.f.	denominator d.f.	*F*	*p*	estimate	lower 95% CI	upper 95% CI	*t*
metabolic traits	mass	1	38.20	2.517	0.121	412.47	−113.75	938.69	1.587
	SMR	1	38.84	8.881	0.005	−329.98	−553.98	−105.98	−2.980
	AS	1	38.00	0.668	0.419	−212.03	−737.32	313.27	−0.817
	EPOC	1	38.70	10.693	0.002	−275.84	−446.49	−105.18	−3.270
swimming performance	length	1	61.74	0.230	0.633	17.36	−54.97	89.69	0.480
	*U*_gt_	1	61.74	15.241	<0.001	−1826.57	−2760.42	−890.73	−3.904
	*U*_max_	1	61.78	4.733	0.033	−480.70	−922.40	−38.98	−2.176

## 4. Discussion

Vulnerability to harvest, such as capture by trawl, is likely a complex, integrative characteristic influenced by several morphological and behavioural traits [13,25]. The ecological and evolutionary importance of intraspecific variation in phenotypes (e.g. animal personality) has been the focus of a large body of recent literature, and several studies have begun to highlight the importance of behavioural traits within the context of harvest vulnerability [13,17,18]. This study shows for the first time that physiological traits also play a key role in determining which individuals are captured during trawling and which are not. Fish displayed repeatable, intraspecific variation in vulnerability, directly influenced by anaerobic capacity and swimming ability, with additional links to aerobic swimming performance and SMR. The fact that some fish are consistently more prone to capture suggests that suites of traits that influence overall vulnerability to trawling could be selected by harvest-associated selection when fishing pressure is high [10,14,44,45].

Fish that were least vulnerable to capture had a higher anaerobic capacity and burst swimming performance compared with fish that were more catchable. During swimming at the mouth of an oncoming trawl—the scenario that was simulated in this study—fish have been shown to display burst-type anaerobic swimming as they attempt to maintain station ahead of the net [23,26]. The importance of anaerobic performance on the ability to escape capture by trawling presents interesting parallels with the anaerobic locomotory activity employed by fish during escapes from natural predators [40]. Indeed, similarities have previously been drawn between the economics of deciding whether to stay versus flee when avoiding both predators and human-associated disturbances such as trawls [25,41], but the results here indicate a direct link between vulnerability and physiological traits related to locomotion. The consequences of potential selection on anaerobic capacity from large-scale fisheries are difficult to predict, but could include selection for faster burst-type swimming speeds, and, potentially elevated metabolic rates, given the link between SMR and total time spent in the net. Although these results suggest that harvest-induced selection and natural selection can sometimes act in the same direction, it is important to note that the intensity of the selection induced by humans and natural predators can differ substantially [3], depending on several factors including the magnitude of natural versus human-induced mortality and the life-stage at which mortality occurs [2]. A promising avenue for future research will be understanding interactions between harvest-induced and natural selection, even when these forces seemingly act in parallel.

Aerobic swimming performance and AS were less important for vulnerability relative to anaerobic performance. When included in the LME, fish with a higher AS were less catchable, but this trend was not significant. Water speed in the simulated trawl was 38 cm s^−1^, which was beyond *U*_gt_ for most fish in the study, and so it is perhaps not surprising that AS did not directly influence vulnerability. Similarly, the speed of an actual trawl is purposely set to be beyond the sustained swimming ability of targeted fish, in an attempt to force them to engage in burst-type anaerobic swimming [25]. Still, aerobic swimming ability may be linked to vulnerability, given the association observed between *U*_gt_ and time spent in the net. A likely scenario is that anaerobic traits have the strongest direct effect on vulnerability during trawling but, due to correlations among traits (electronic supplementary material, table S2), the least catchable fish are also among those with the greatest aerobic swimming performance. Additional research could examine how variation in trawl speed or duration affects the importance of AS in vulnerability by trawl [46], or how abiotic factors such as oxygen availability or temperature might also play a role.

Overall, fish with a higher SMR were also less catchable. Baseline metabolic rate is a partially heritable trait that shows wide, repeatable intraspecific variation [47]. In fishes, SMR has been shown to influence demand for food and oxygen and is related to various aspects of predator avoidance [48,49], which could include avoidance of fishing gear. However, considering this study only examined direct interactions between the fish and the trawl mouth (beyond the point where an early fleeing response could be initiated [25,50]), the effect of SMR on vulnerability was to some degree unexpected. Again, it is possible that the link between SMR and time in the net stemmed from correlations with anaerobic traits (electronic supplementary material, table S2) that primarily determine an individual's vulnerability to capture by trawling. However, even correlated selection for a higher SMR by trawling could have profound implications for wild populations. Among individuals, SMR has links with growth rate that vary with context—in high food environments, SMR tends to be associated with increased growth, but, when food availability is limiting, there is generally decreased growth within a population [47,51]. Historical selection for high SMR in food-limiting ecosystems could partially explain the altered growth trajectories and reproductive investment observed in contemporary fisheries stocks [44]. An important avenue for future research will be to compare physiological traits, such as SMR, between populations of the same species that live in similar habitats but have experienced varying degrees of harvest pressure.

While small-scale simulations with representative species provide a valuable foundation for understanding the physiological basis of harvest-associated selectivity and its evolutionary consequences [18], there is the question of how the findings from such studies may be applied to actual fisheries procedures. Swim flumes have previously been used to study the behaviour of fish in response to trawling procedures [24,52,53], and here we used forced swimming to emulate the tendency of fish to hold station in relation to an approaching trawl net [26]. The chosen water current speed was, in relative terms, similar to that which would be used in a trawl with larger commercial species, in that it was at the upper boundary of the sustained swimming speed of the targeted fish [25]. One important difference between our simulation and actual trawls is the number of fish involved in the trawling scenario. Commercial trawls may target shoals consisting of thousands of individuals, while in our study, we were limited in the numbers of fish we could use per trial because increased numbers of fish make it more difficult to quantify behaviours of individuals. With more fish in a trawl, the importance of interactions with other individuals may become elevated. Furthermore, our simulation represents the response of fish to a looming trawl under well-lit conditions. Under very low light levels, fish may show a reduced avoidance response and be more easily swept into the trawl [26], perhaps reducing the importance of intraspecific variation in performance for influencing vulnerability. In large-scale trawls, fish can also have additional opportunities to escape capture, which would also involve anaerobic swimming, such as penetrating through the net mesh or panels designed to reduce bycatch. Similarly, in an actual trawl, the opportunity for escape will be influenced by the location of the fish relative to the edge, top, or bottom or the trawl. Fish closer to the centre may have a decreased likelihood of escape, but even in this scenario, an increased anaerobic capacity could allow burst-type swimming that could allow the fish to move laterally while maintaining a forward vector to stay ahead of the oncoming net.

In summary, vulnerability to trawling is a repeatable characteristic of individual fish linked directly to anaerobic swimming ability, and likely indirectly to aerobic swimming ability and baseline metabolic rate. It is therefore entirely possible that commercial trawl fisheries may select for these traits, thus generating a mechanism by which FIE could alter the physiological traits of fish within a population. These results also have important implications for understanding the impacts of harvest on the physiological phenotypes of commercially exploited animals, therefore providing insight into the mechanisms of evolutionary effects, stock resiliency and the ability of fishes to respond to environmental change [44,51,54]. This is a useful starting point, but more information is needed on the physiological underpinnings of harvest-induced selection [13,21,44].

## Data accessibility

The datasets supporting this article have been uploaded as part of electronic supplementary material, table S1.
